# Effect of eight-week high-intensity interval training versus moderate-intensity continuous training programme on body composition, cardiometabolic risk factors in sedentary adolescents

**DOI:** 10.3389/fphys.2024.1450341

**Published:** 2024-08-09

**Authors:** Fucheng Sun, Craig A. Williams, Qiang Sun, Feng Hu, Ting Zhang

**Affiliations:** ^1^ Department of Physical Education, Faculty of Social Science, Nanjing Agricultural University, Nanjing, China; ^2^ Children’s Health and Exercise Research Centre, Public Health and Sports Sciences, University of Exeter Medical School, Faculty of Health and Life Sciences, University of Exeter, Exeter, United Kingdom; ^3^ Sport Science Research Institute, Nanjing Sport Institute, Nanjing, China; ^4^ Hospital, Nanjing Agricultural University, Nanjing, China

**Keywords:** sprint interval training, adolescent, cardiovascular disease, visceral fat, waist circumference, clinical biomarker

## Abstract

**Objectives:**

This study aimed to assess and compare the effect of an 8-week high-intensity interval training (HIIT) or moderate-intensity continuous training (MICT) programme on body composition and cardiovascular metabolic outcomes of sedentary adolescents in China.

**Methods:**

Eighteen sedentary normal-weight adolescents (age: 18.5 ± 0.3 years, 11 females) were randomized into three groups. HIIT group protocol consisted of three sessions/week for 8-week of “all out” sprints to reach 85%–95% of HR_max_, and MICT group protocol undertook three sessions/week for 8-week of continuous running to reach 65%–75% of HR_max_. The control group resumed normal daily activities without any intervention. Blood pressure and body composition were measured, and fasting blood samples were obtained at baseline and 48 h post-trial. Mixed-design ANOVA analysis was employed followed by *post hoc* t-tests and Bonferroni alpha-correction was used to evaluate interaction, between-group, and within-group differences, respectively.

**Results:**

Results indicated that HIIT and MICT similarly affected body fat mass (*p* = 0.021, ES = 0.19; *p* = 0.016, ES = 0.30, respectively), body fat percentage (*p* = 0.037, ES = 0.17; *p* = 0.041, ES = 0.28, respectively), visceral fat area (*p* = 0.001, ES = 0.35; *p* = 0.003, ES = 0.49, respectively) of body composition. A positive outcome was observed for waist/hip ratio (*p* = 0.033, ES = 0.43) in HIIT, but not MICT (*p* = 0.163, ES = 0.33). No significant differences were found between groups for any clinical biomarkers. However, pairwise comparison within the group showed a significant decrease in systolic blood pressure (*p* = 0.018, ES = 0.84), diastolic blood pressure (*p* = 0.008, ES = 1.76), and triglyceride (*p* = 0.004, ES = 1.33) in HIIT, but no significant differences were found in the MICT and Control group.

**Conclusion:**

Both 8-week HIIT and MICT programmes have similar positive effects on reducing body fat mass, fat percentage, and visceral fat area. However, sedentary adolescents may have limited scope to decrease insulin resistance after these 8-week interventions. Notably, the 8-week HIIT intervention was highly effective in increasing cardiometabolic health compared to the MICT. The exercise intensity threshold value and metabolic outcomes of high-intensity interval sprints should be explored further to extend the long-term benefit in this cohort.

## Introduction

Cardiovascular-related chronic diseases, e.g., obesity, type 2 diabetes, and induced arterial stiffness are a major global health concern. The cause of chronic diseases is multifactorial but ultimately results from a chronic imbalance of metabolism (Sharifi-Rad et al., 2020; Raut and Khullar, 2023). Waist circumference, fasting glucose, triglycerides, low-density lipoprotein cholesterol, insulin resistance, and chronic inflammation have all been implicated in the development of these diseases and are components of the metabolic syndrome (Kessler et al., 2012; Gottsäter et al., 2015; Palombo and Kozakova, 2016). The origins of these chronic diseases often lie in childhood or adolescence (Cockcroft et al., 2018), whereas active lifestyle and habits developed very early in childhood and adolescence often lead into positive habits in adulthood (Telama et al., 2014). Furthermore, if obesity becomes established in adolescence, a later spontaneous reversal of the weight gain is uncommon (Thompson et al., 2007; Patton et al., 2011; Brisbois et al., 2012), and highlights the importance of physical activity intervention for this young cohort. Furthermore, a considerable number of the investigated health outcomes had a significant association with overweight or obese children and adolescent cohorts (Delgado-Floody et al., 2018; Dias et al., 2018; Espinoza-Silva et al., 2019), and whilst the majority of children and adolescents are typically normal-weight, yet still exposed to potential risk factors, e.g., increased lipid levels and high weight category, they are still likely to demonstrate cardiovascular disease later in adulthood (Rosengren et al., 2017; Cooper and Radom-Aizik, 2020; Agbaje, 2024). Therefore, considering that cohorts of 18-year-olds will regularly spend several years in a constant environment, i.e., college or university, this presents a perfect opportunity to intervene effectively before later adult years set in. Hence, strategies that can modify glucose metabolism, insulin resistance, and visceral adiposity during youth may play an important role in disease prevention in later life (Esquivel Zuniga and DeBoer, 2021; Calcaterra et al., 2022; Polidori et al., 2022).

Higher levels of exercise are regularly prescribed for the prevention and treatment of several cardiovascular diseases, e.g., type 2 diabetes (De Nardi et al., 2018; Syeda et al., 2023), arterial stiffness (Way et al., 2019; Sequi-Dominguez et al., 2023), and stroke (Boehme et al., 2017; Gjellesvik et al., 2021). Children and adolescents are currently recommended to undertake at least 60 min of moderate-to-vigorous physical activity on a daily basis, but 81% of adolescents do not meet the WHO global recommendations on physical activity for health (Chaput et al., 2020; Farooq et al., 2020; Marzi et al., 2022). Moderate-intensity continuous training (MICT) has been shown to provide numerous health benefits, including improved cardiorespiratory fitness (Faria et al., 2020), reduced body fat (Mendonça et al., 2022), and enhanced cardiovascular function (Collins et al., 2023), making it a cornerstone of many physical activity guidelines for children and adolescents. However, MICT requires a longer duration of continuous exercise, which may pose challenges for adherence among children and adolescents with busy schedules or lower motivation levels, potentially affecting their long-term commitment to regular physical activity. Consequently, researchers have focused on alternative forms of exercise, i.e., high-intensity interval training (HIIT), which is considered time-efficient and enjoyable. HIIT may induce similar or greater potential benefits compared with MICT in children and adolescents (Corte de Araujo et al., 2012; Martin-Smith et al., 2020). As a result, HIIT has emerged as a promising alternative, potentially more appealing and practical for youth due to its shorter duration and varied intensity. The mechanisms by which HIIT produces beneficial effects are complex. However, physiologists generally agree that, compared to MICT, HIIT effectively enhances lipid metabolism (Gripp et al., 2021), levels of anti-inflammatory factors (Khalafi and Symonds, 2020), insulin signaling pathways (Islam and Gillen, 2023), and endothelial nitric oxide synthase expression (Khalafi et al., 2022). These improvements contribute to the prevention of metabolic disorders and the amelioration of adverse cardiometabolic outcomes. In particular, HIIT has been shown to improve body composition, aerobic fitness, and vascular function during supervised lab-based studies (Barker et al., 2014; Dias et al., 2018; Ingul et al., 2018), which is associated with a reduced risk of cardiovascular events in later life. However, adherence, enjoyment, and health benefits of school-based field HIIT performed independently are yet to be fully understood, e.g., the enjoyment levels observed in supervised, structured settings may not necessarily translate to independent exercise sessions, and the health benefits may not be fully replicated in real-world environments (Lubans et al., 2021; Sun, 2024). In addition, previous studies have focused more on the relationship between overweight and metabolic health in adolescents (Racil et al., 2016; Miguet et al., 2020). Although laboratory-based HIIT studies have played an important role in consultation and clinical treatment, the current situation is that sedentary behavior and a lack of exercise are prevalent in young people (Arundell et al., 2016; Reilly et al., 2022).

Characterizing the effects of two different intervention protocols (i.e., HIIT vs. MICT) may help individuals to find the most appropriate approach for targeting academic performance and future well-being. The research in this cohort may also improve the comprehension of school/college administrators about the optimal exercise strategies to implement. Therefore, the purpose of this study was two-fold: i) to analyze the within-group variations of HIIT and MICT interventions lasting 8 weeks on body composition and cardiovascular metabolic of sedentary adolescents; and ii) to analyze the between-group differences of both training interventions on body composition and cardiovascular metabolic outcomes of sedentary adolescents. The primary hypothesis of this study was that HIIT and MICT will significantly improve body composition in terms of body mass index, fat mass percentage, visceral fat area, and waist/hip ratio in sedentary youth compared to a control group performing regular physical activity intervention. The secondary hypothesis was that cardiovascular metabolic outcomes benefits in terms of blood pressure, total cholesterol, triglyceride, low-density lipoprotein cholesterol, and HOMA-IR would decrease in both intervention groups compared to the control group.

## Methods

### Study participants

**FIGURE 1:**
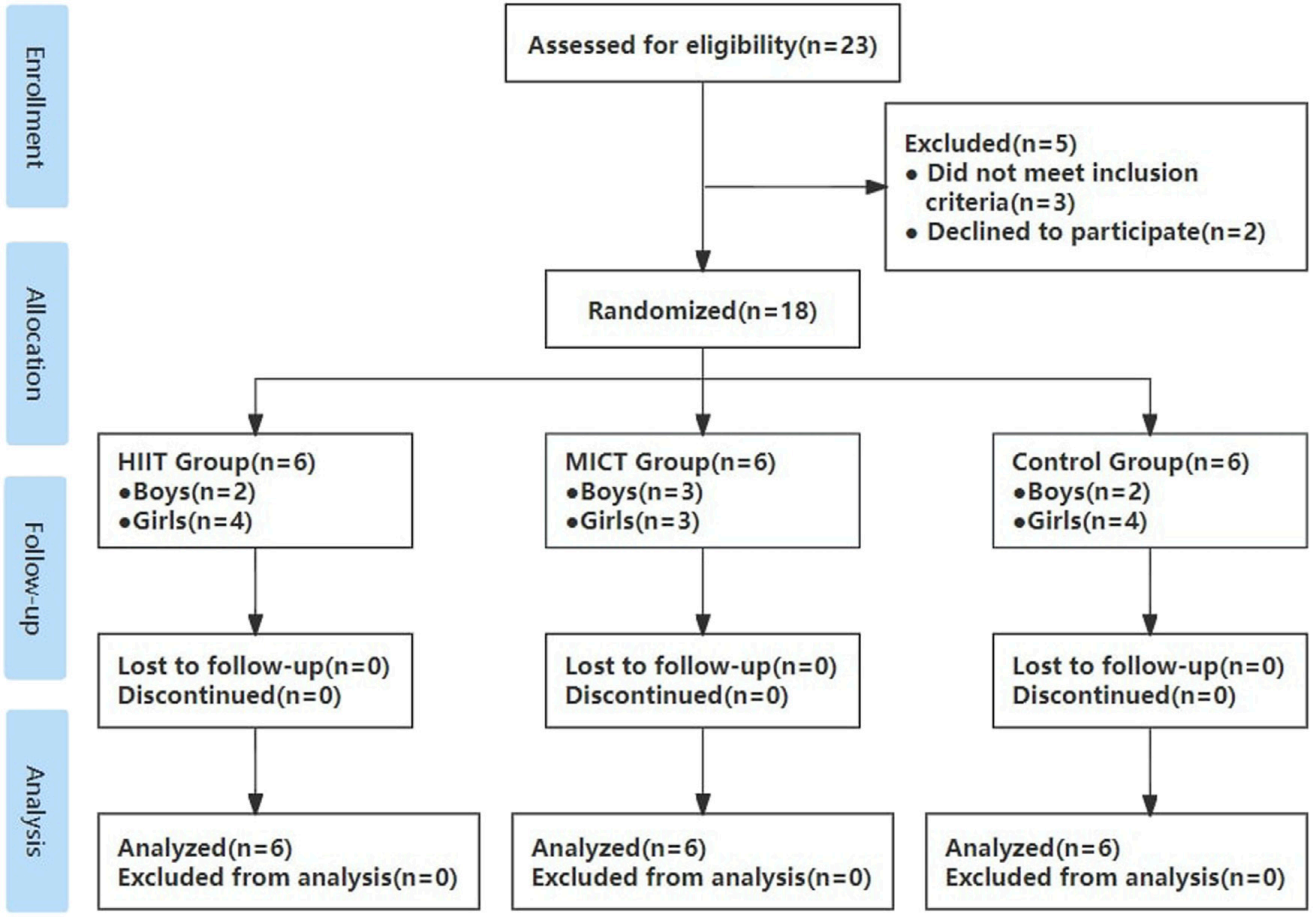
Flow diagram throughout the course of the study. HIIT indicates high-intensity interval training; MICT indicates moderate-intensity continuous training; Control indicates no intervention.

A power analysis completed with G*Power (ver. 3.1.9.7; Heinrich-Heine-Universität, Düsseldorf, Germany) using a medium effect size revealed that 24 participants would be necessary to detect a significant medium effect (d = 0.5) for the outcome in a within-between interaction with alpha error probability set at 0.05 and power adjusted to 0.80. However, the COVID-19 pandemic made enrollment and fidelity to aspects of study protocols challenging (McDermott and Newman, 2020). In this trial, pandemic-related restrictions that limited in-person visits resulted in unprecedented obstacles to interviewing potential participants, trial enrollment, data collection, and intervention delivery for this trial. Eventually, twenty-three healthy normal-weight youth were recruited from Nanjing Agricultural University, Nanjing, China. While a disappointing result for the exceptional time and effort placed into enrollment, this trial also remains a helpful guide to Chinese sedentary adolescents towards finding ways of engaging appropriate protocols and improving cardiometabolic health outcomes. Importantly, it should be noted that the sample was highly representative of the study population, as exclusion criteria included any known disease or contraindications to exercise and the use of medications or substances known to influence blood pressure, cholesterol, and carbohydrate metabolism. Participants were inactive as assessed by participating in television, computer, smartphone, video games viewing, and sitting socializing for over 8 h daily in the past 5 months. Furthermore, participants took China’s college entrance exam before recruitment, had no regular physical activity during the last 3 years, and performed only activities of daily living. After participants underwent an initial health-related behavior questionnaire testing and electrocardiogram examination, one female participant was excluded because of bradycardia, and two male participants failed to join the project because of their enrolment in more than 3 h per week structured program of physical activity recently, one male and one female participant withdrew from the project due to personal reasons unrelated to the experiment. Thus, a total of 18 participants (age: 18.5 ± 0.3 years, 11 females) completed this investigation from November 2021 to January 2022 (Figure 1). This investigation was conducted following the recommendations of the Declaration of Helsinki for Human Studies and the university ethical approval (number: RT-2023-01). Written informed consent was obtained from all participants.

### Body composition and blood pressure measurements

Multifrequency bioelectrical impedance analysis of body composition is regarded as an appropriate alternative to dual-energy x-ray absorptiometry, and is widely used in clinical practice (Anderson et al., 2012). Body mass index (BMI), body fat mass (BF), body fat percentage (BFP), visceral fat area (VFA), waist/hip ratio (WHR) were evaluated before and after intervention using multifrequency bioelectrical impedance analysis (Multifrequency bioelectric impedance analyzer InBody 720, Biospace Co. Ltd., Seoul, Korea). All assessments were conducted between 9 a.m. and 12 p.m. to minimize variability due to time-of-day effects or physical activity. Measurements are performed on five segments (right upper limb, left upper limb, trunk, right lower limb, and left lower limb) using six bioimpedance frequencies (including 1 kHz, 5 kHz, 50 kHz, 250 kHz, 500 kHz, and 1,000 kHz). Participants wore everyday indoor clothing and were required to stand barefooted in an upright position with their feet on the feet electrodes of the machine platform and their arms abducted with hands gripping onto the hand electrodes of the handles during the test.

Blood pressure was determined using an auto-inflating cuff (Omron, HEM-1020, Dalian, China). To ensure consistency, all measurements were taken from 9 a.m. to 12 p.m. on the same day, following a 10-min rest period. Participants were seated, with their back supported, feet on the ground, and arms supported at heart level. Measurements were taken on the right arm, and the average of three consecutive readings was recorded. This standardized protocol helped to minimize variability due to time-of-day effects and participant posture. Participants were also advised to remain silent throughout the measurement.

### Biochemical measurements

Blood samples were collected from the participants’ peripheral veins in the morning after a fasting period of 12 h. The whole blood samples were centrifuged at 3,500 revolutions per min for 8 min. Plasma was separated and stored at −80°C until assayed. The automated biochemical analyzer (ARCHITECT c16000, Abbott, Singapore) and the automated chemiluminescence immunoassay analyzer (i 2000SR, Abbott, Singapore) were used to detect the biochemical indicators. Plasma glucose (PG), total cholesterol (TC), triglyceride (TG), high-density lipoprotein cholesterol (HDL-C), and low-density lipoprotein cholesterol (LDL-C) were determined by Hexokinase Method, COD-PAP, GPO-PAP, CAT-Assay, and Surfactant Assay respectively, using Biosino Kits (Biosino Bio-technology and science INC., Beijing, China). Plasma insulin was determined by CLIA, using an Abbott Insulin Reagent Kit (DENKA SEIKEN Co., LTD., Tokyo, Japan). Hypersensitive C-reactive protein (Hs-CRP) was determined by Immunoturbidimetry, using an Abbott CPR Vario (DENKA SEIKEN Co., LTD., Tokyo, Japan). All detection steps and operations were carried out in accordance with the kit instructions. Insulin resistance was assessed using the homeostasis model assessment of insulin resistance (HOMA-IR) according to the formula: HOMA-IR = fasting plasma insulin × fasting plasma glucose/22.5 (Invitti et al., 2003).

### Interventions

**FIGURE 2:**
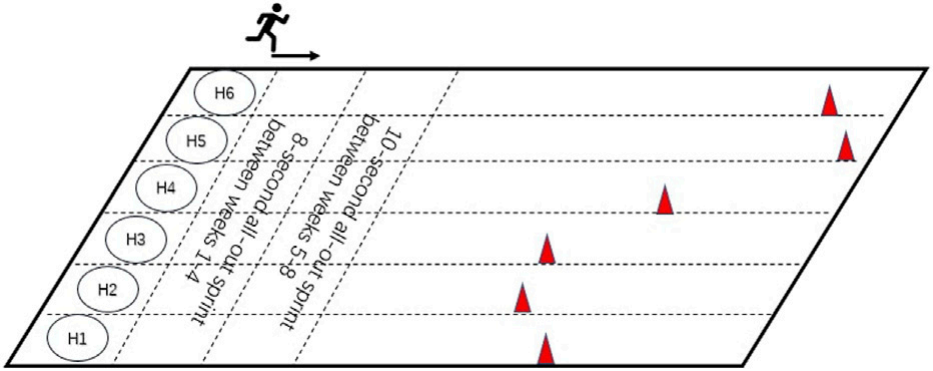
Schematic chart of high-intensity interval training path.

Participants were randomly allocated into three groups: HIIT (n = 6), MICT (n = 6), and Control (n = 6). The intervention of HIIT group was conducted three times per week, 30 min per session (including a 5-min warm-up, 20-min sprint interval training, and a 5-min cooldown and stretching) for 8 weeks, whilst the MICT group received three times per week, 30 min per session (including a 5-min warm-up, and 20-min continuous running, and a 5-min cooldown and stretching) for 8 weeks, Control group was advised to maintain their current physical activity levels and dietary habits during the study. All participants were recommended to maintain a daily calorie intake appropriate for late adolescents, depending on body size and activity level, and to avoid unhealthy snacks. HIIT group consisted of three sessions/week for 8 weeks of “all out” sprint, 8-s sprint, and 24-s active recover/during 1–4 weeks and 10-s sprint and 30-s active recover/during 5–8 weeks. HIIT group participants were required to turn-back and walk after every all-out sprint running and repeat the exercise. Participants were required to try to reach the location of the cone within the specified time. HIIT group intensity was set at 85%–95% of HR_max_ with active recovery periods of walking between bouts, MICT group intensity was set at 65%–75% of HR_max_. HR_max_ was determined through a baseline VO_2max_ test using the Bruce protocol until the point of voluntary exhaustion. This test was performed on a treadmill (H/P/Cosmos para graphics, H/P/COSMOS Sports and Medical, Germany). Simultaneously, respiration and heart rate were monitored using a wearable metabolic system (K5 Wearable Metabolic System, COSMED, Italy). HIIT group exercise interventions were performed on a short track of Nanjing Agricultural University Athletics Field (Figure 2), MICT group interventions were performed on the circle track of the same athletics field. The HIIT group completed between 21-24 all-out sprint running within a 20-min session. The total distance of the HIIT group in each session was 4.20 ± 0.56 km, and the total distance of the MICT group was 3.98 ± 0.26 km. The intensity of all the intervention groups was monitored by the POLAR OH1 MODEL 2L (Patented WR3, Malaysia). All interventions were also supervised by professionals, with intensity feedback and speed adjustments using Polar monitors with GPS tracking, recording heart rate every second. Researchers also encouraged the participants to elicit the running at all-out intensity during HIIT programs, the heart rate response during HIIT programs ranged from 96 to 197 bpm (113 ± 8.62 bpm-189 ± 5.38 bpm), including the lowest heart rates recorded during the warm-up phase. Participants were encouraged to run and avoid any walking during MICT programs, the heart rate response during MICT programs ranged from 90 to 188 bpm (103 ± 8.23 bpm-180 ± 6.79 bpm), also accounting for warm-up periods. The heart rates generally corresponded to the target intensity during the main exercise bouts.

As shown in Figure 2, the location of the cones is determined by each participant, e.g., 8-s all-out sprint distance between weeks 1–4, and 10-s all-out sprint distance between weeks 5–8. The cones were repositioned by a professional supervisor at the first training session of each week to avoid deviation due to training fitness increase.

### Statistical analysis

The descriptive characteristics of the participants are presented as mean and SD using the IBM SPSS Statistics version 26.0 (Chicago, IL, United States). Normality and homogeneity of the sample were preliminary tested using Shapiro-Wilk test and Levene’s test, respectively (Razali and Wah, 2011). The normality of the outcomes was explored individually and for each group revealing a *p* > 0.05 for all the outcomes considered. The homogeneity of the outcomes was tested using Levene’s presenting values above *p* > 0.05. One-way ANOVA and Bonferroni’s *post hoc* tests were used for the evaluation baseline data of three groups. A mixed model ANOVA with group (CON, MICT, HIIT) and time (baseline and after intervention) analyzed the effects of body composition and cardiometabolic outcomes after the intervention. Interaction was reported in the format of *p*-value and partial eta squared (η^2^_p_). An η^2^_p_ of 0.01 indicated a small effect, 0.06 a medium effect, and 0.14 a large effect (Lakens, 2013). A simple effect test was conducted after confirmation of interaction (group × time) to avoid misleading analyses. Magnitude of standardized differences between two means were evaluated using the standardized effect size of Cohen’s d (ES), with the equation (mean post−mean pre)/SD pooled. An ES of 0.20, 0.50, and 0.80 was considered to represent a small, moderate, and large change between means (Kazis et al., 1989). A level of *p* < 0.05 was set *a priori* to establish statistical significance.

## Results

A total of 18 adolescents (age = 18.5 ± 0.3 years) performed the baseline assessments. The baseline characteristics of the study participants are shown in Table 1. There was a significant difference between HIIT and CON in BM and BMI (*p* < 0.05), but no other significant differences were observed between the groups before exercise intervention (*p* > 0.05). Furthermore, no testing or training-related severe injuries occurred over the trial period, and the attendance rates were 100% for the two intervention groups, i.e., HIIT and MICT.

**TABLE 1:** Body composition before and after training for HIIT, MICT, and Control groups.

Outcomes	Group	Pretraining	Trained	ANOVA (F, p, η^2^_p_)								
				Time effect			Group effect			Time x group effect		
BM (kg)	HIITMICTControl	64.4 ± 5.161.3 ± 6.453.5 ± 8.5	63.8 ± 4.560.6 ± 5.953.4 ± 8.0	2.21	0.158	0.13	4.25	0.035^*^	0.36	0.45	0.648	0.06
SM (kg)	HIIT MICT Control	25.7 ± 4.6 24.6 ± 3.7 21.5 ± 4.8	26.3 ± 4.3 24.9 ± 3.2 21.7 ± 4.3	3.59	0.078	0.19	1.76	0.206	0.19	0.54	0.593	0.07
BF (kg)	HIIT MICT Control	17.8 ± 5.1 16.4 ± 3.7 13.9 ± 5.1	16.8 ± 5.2 15.4 ± 3.2 13.7 ± 4.7	11.35	0.004^#^	0.43	0.93	0.418	0.11	1.45	0.266	0.16
BMI (kg/m2)	HIIT MICT Control	23.5 ± 2.3 22.5 ± 1.7 20.3 ± 1.7	23.4 ± 2.3 22.2 ± 1.6 20.3 ± 1.7	1.99	0.179	0.18	4.36	0.032^*^	0.37	0.50	0.618	0.06
BFP (%)	HIIT MICT Control	27.8 ± 8.7 26.8 ± 5.7 26.0 ± 8.2	26.3 ± 8.6 25.3 ± 4.8 25.6 ± 7.1	9.05	0.009^#^	0.38	0.05	0.949	0.01	0.82	0.460	0.10
VFA (cm2)	HIIT MICT Control	76.4 ± 27.5 67.8 ± 18.8 57.1 ± 27.9	67.1 ± 25.2 59.9 ± 13.0 55.7 ± 25.1	23.92	<0.001^#^	0.62	0.65	0.538	0.08	3.65	0.051	0.33
WHR	HIIT MICT Control	0.84 ± 0.03 0.83 ± 0.03 0.82 ± 0.02	0.83 ± 0.04 0.82 ± 0.02 0.83 ± 0.02	1.84	0.195	0.11	0.14	0.869	0.02	3.99	0.041^*^	0.35

Results shown as mean ± SD.

Abbreviations: HIIT, high-intensity interval training; MICT, moderate-intensity continuous training; BM, body mass; SM, skeletal muscle; BF, body fat mass; BMI, body mass index; BFP, body fat percentage; VFA, visceral fat area; WHR, waist/hip ratio. *p < 0.05, #*p* < 0.01.

**FIGURE 3:**
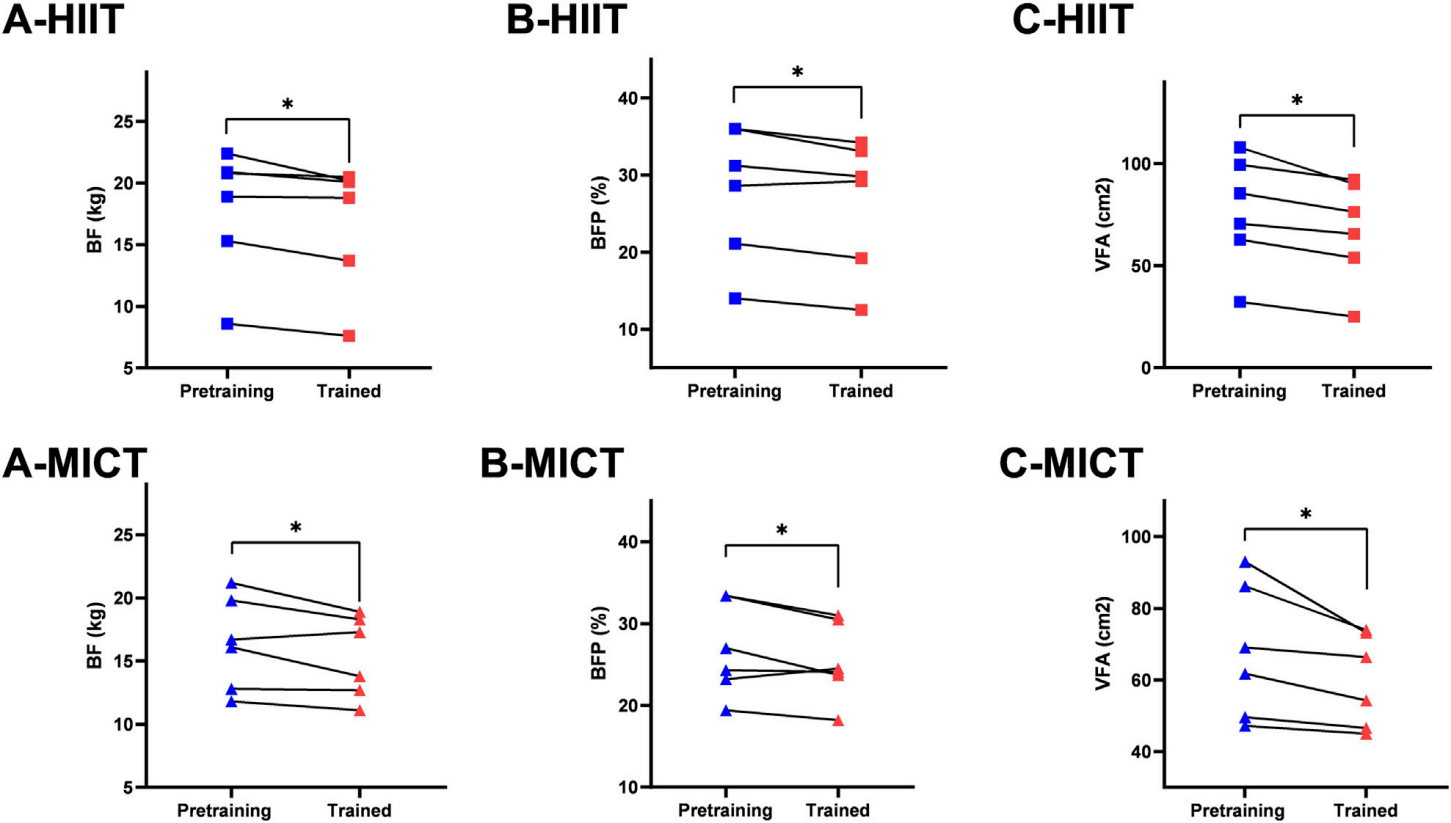
Within-group comparison in (A) BF (body fat mass); (B) BFP (body fat percentage); (C) VFA (visceral fat area). * Indicates significant differences within-group comparisons (*p* < 0.05).

**FIGURE 4:**
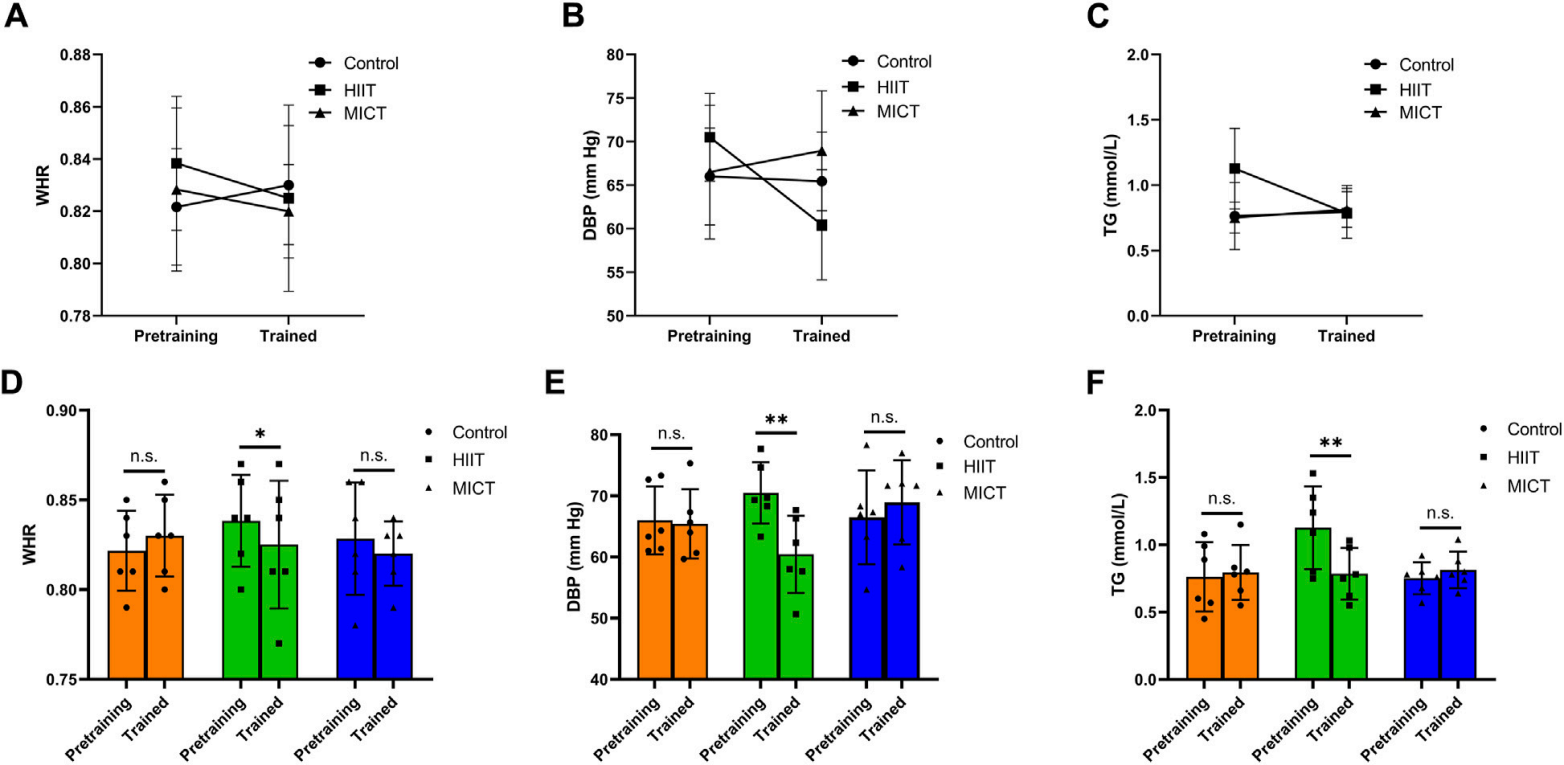
Group × time significant interactions effect in **(A)** WHR (waist/hip ratio); **(B)** DBP (diastolic blood pressure); **(C)** TG (triglyceride). Pre- and post-training values [Mean (±SD)] for **(D)** WHR, **(E)** DBP, and **(F)** TG in HIIT (High-intensity interval training), MICT (Moderate-intensity continuous training), and Control groups. * Indicates significant differences within-group comparisons (*p* < 0.05), * * Indicates significant differences within-group comparisons (*p* < 0.01).

Descriptive statistics of pre- and post-intervention values of body composition can be found in Table 1. There was time main effect of BF (*p* = 0.004, η2_p_ = 0.43), BFP (*p* = 0.009, η2_p_ = 0.38), VFA (*p* < 0.001, η2_p_ = 0.62), no significant differences across the two-time point in BM (*p* = 0.158, η2_p_ = 0.13), SM (*p* = 0.078, η2_p_ = 0.19), BMI (*p* = 0.179, η2_p_ = 0.12), and WHR (*p* = 0.195, η2_p_ = 0.11). There was group main effect of BM (*p* = 0.035, η2_p_ = 0.36), BMI (*p* = 0.032, η2_p_ = 0.37), no significant differences between groups in SM (*p* = 0.206, η2_p_ = 0.19), BF (*p* = 0.418, η2_p_ = 0.11), BFP (*p* = 0.949, η2_p_ = 0.01), VFA (*p* = 0.538, η2_p_ = 0.08), and WHR (*p* = 0.869, η2_p_ = 0.02). Figure 3 presents the within-and between-group variations for the body composition. The within-group changes revealed a significant decrease in BF in HIIT (*p* = 0.021, ES = 0.19) and MICT (*p* = 0.016, ES = 0.30), whereas no significant changes occurred in CON (*p* = 0.585, ES = 0.04). Significant decrease in BFP in HIIT (*p* = 0.037, ES = 0.17) and MICT (*p* = 0.041, ES = 0.28), whereas no significant changes occurred in CON (*p* = 0.499, ES = 0.06). Significant decrease in VFA in HIIT (*p* = 0.001, ES = 0.35) and MICT (*p* = 0.003, ES = 0.49), whereas no significant changes occurred in CON (*p* = 0.527, ES = 0.06). Between-group variations revealed a significant difference in post-interventions between HIIT and CON (*p* = 0.036, ES = 1.60) in BM and between HIIT and CON (*p* = 0.037, ES = 1.53) in BMI, however considering that there were also significant differences of the baseline between HIIT and CON (*p* = 0.041, ES = 1.56) in BM and between HIIT and CON (*p* = 0.031 ES = 1.62) in BMI, the statistically significant is severely limited. There was also a group × time significant interactions effect in WHR (*p* = 0.041, η2_p_ = 0.35), and a trend of significant interactions effect in VFA (*p* = 0.051, η2_p_ = 0.33), although the result was not significant. However, no significant interaction effects were found in BM (*p* = 0.648, η2_p_ = 0.06), SM (*p* = 0.593, η2_p_ = 0.07), BF (*p* = 0.266, η2_p_ = 0.16), BMI (*p* = 0.618, η2_p_ = 0.06), and BFP (*p* = 0.460, η2_p_ = 0.10). Follow up for this interaction indicated that there were no significant differences between groups at baseline and post-intervention in WHR. However, a pairwise comparison within the group showed that HIIT group had a significant decrease (*p* = 0.033, ES = 0.43) in WHR, and no significant difference was found in MICT (*p* = 0.163, ES = 0.33) and Control group (*p* = 0.163, ES = 0.37) (Figure 4).

Descriptive statistics of pre- and post-intervention values of clinical biomarkers can be found in Table 2. No significant differences in clinical biomarkers were found between groups in baseline and post-intervention (*p* > 0.05). There were no significant differences in clinical biomarkers within groups in baseline and post-intervention in HIIT and MICT. However, PG had a time main effect in CON (*p* = 0.008, η^2^_p_ = 0.39). The within-group changes revealed a significant increase in PG in CON (*p* = 0.024, ES = 0.64), whereas no significant changes occurred in HIIT (*p* = 0.059, ES = 0.63) and MICT (*p* = 0.443, ES = 0.08). Significant interactions were found between groups and time (pre-post) in the mixed ANOVA conducted for DBP (*p* = 0.041; η^2^_p_ = 0.35) and TG (*p* = 0.023; η^2^_p_ = 0.40) (Figure 4). No significant interactions were found in mixed ANOVA for the case of SBP (*p* = 0.134; η^2^_p_ = 0.24), PG (*p* = 0.472, η^2^_p_ = 0.10), Insulin (*p* = 0.095, η^2^_p_ = 0.27), TC (*p* = 0.423, η^2^_p_ = 0.11), HDL-C (*p* = 0.115, η^2^_p_ = 0.25), LDL-C (*p* = 0.347, η^2^_p_ = 0.13), hs-CRP (*p* = 0.900, η^2^_p_ = 0.01), and HOMA-IR (*p* = 0.141, η^2^_p_ = 0.23). PG in three groups increased (3.91%, 1.40%, and 2.20%, respectively), insulin in HIIT decreased (10.80%), but insulin in MICT and control both increased (19.65% and 49.54%, respectively). HOMA-IR also showed a similar trend among the three groups, HOMA-IR decreased in HIIT (5.63%), increased in MICT and Control (21.28% and 55.17%, respectively). Following up this interaction indicated that there was no significant difference between groups at baseline and post-intervention in DBP and TG. However, pairwise comparison within the group showed HIIT group, there was a significant decrease in SBP (*p* = 0.018, ES = 0.84), DBP (*p* = 0.008, ES = 1.76), and TG (*p* = 0.004, ES = 1.33), respectively. No significant difference was found in MICT (*p* = 0.932, ES = 0.02) and Control group (*p* = 0.983, ES = 0.01) for SBP, MICT (*p* = 0.466, ES = 0.34) and Control group (*p* = 0.867, ES = 0.10) for DBP, and MICT (*p* = 0.552, ES = 0.48) and Control group (*p* = 0.759, ES = 0.14) for TG.

**TABLE 2:** Clinical biomarkers before and after training for HIIT, MICT, and Control groups.

Outcomes	Group	Pretraining	Trained	ANOVA (F, p, η^2^_p_)								
				Time effect			Group effect			Time × group effect		
SBP (mmHg)	HIIT MICT Control	114 ± 6.3 115 ± 9.5 109 ± 8.8	108 ± 9.6 115 ± 7.5 109 ± 10.3	2.48	0.136	0.14	0.80	0.470	0.10	2.31	0.134	0.24
DBP (mmHg)	HIIT MICT Control	71 ± 5.0 67 ± 7.7 66 ± 0.6	60 ± 6.3 69 ± 6.9 65 ± 5.7	2.08	0.170	0.12	0.40	0.680	0.05	3.99	0.041^*^	0.35
PG (mmol/L)	HIIT MICT Control	4.85 ± 0.32 5.22 ± 0.92 4.87 ± 0.38	5.04 ± 0.29 5.29 ± 0.82 5.11 ± 0.35	9.48	0.008^#^	0.39	0.54	0.591	0.07	0.79	0.472	0.10
Insulin (uU/mL)	HIIT MICT Control	9.54 ± 3.37 8.18 ± 2.31 6.09 ± 1.90	8.51 ± 2.41 9.79 ± 2.99 9.11 ± 4.21	2.82	0.114	0.16	0.60	0.561	0.07	2.77	0.095	0.27
TC (mmol/L)	HIIT MICT Control	3.31 ± 0.41 4.06 ± 0.77 3.71 ± 0.44	3.32 ± 0.74 3.99 ± 1.09 3.98 ± 0.48	0.44	0.517	0.03	1.90	0.185	0.20	0.91	0.423	0.11
TG (mmol/L)	HIITMICTControl	1.13 ± 0.310.75 ± 0.120.76 ± 0.26	0.79 ± 0.190.81 ± 0.140.80 ± 0.21	2.00	0.178	0.12	2.06	0.162	0.22	4.91	0.023^*^	0.40
HDL-C (mmol/L)	HIIT MICT Control	1.17 ± 0.14 1.49 ± 0.31 1.51 ± 0.29	1.29 ± 0.19 1.42 ± 0.38 1.59 ± 0.30	1.33	0.267	0.08	2.22	0.143	0.23	2.51	0.115	0.25
LDL-C (mmol/L)	HIIT MICT Control	1.66 ± 0.51 2.16 ± 0.46 1.81 ± 0.18	1.61 ± 0.62 2.10 ± 0.66 1.96 ± 0.33	0.04	0.849	<0.01	1.67	0.221	0.18	1.14	0.347	0.13
Hs-CRP (ug/mL)	HIIT MICT Control	0.90 ± 0.45 0.49 ± 0.22 0.75 ± 0.45	0.70 ± 0.27 0.40 ± 0.46 0.56 ± 0.54	2.49	0.135	0.14	1.50	0.255	0.17	0.11	0.900	0.01
HOMA-IR	HIIT MICT Control	2.03 ± 0.64 1.92 ± 0.64 1.33 ± 0.45	1.92 ± 0.61 2.32 ± 0.82 2.10 ± 1.09	4.27	0.057	0.22	0.63	0.548	0.08	2.24	0.141	0.23

Results shown as mean ± SD.

Abbreviations: HIIT, high-intensity interval training; MICT, moderate-intensity continuous training; SBP, systolic blood pressure; DBP, diastolic blood pressure; PG, plasma glucose; TC, total cholesterol; TG, triglyceride; HDL-C, high-density lipoprotein cholesterol; LDL-C, low-density lipoprotein cholesterol; Hs-CRP, hypersensitive C-reactive protein; HOMA-IR: homeostatic model of insulin resistance. *p < 0.05, #*p* < 0.01.

## Discussion

The primary goal of this study was to evaluate and compare the effects of HIIT and MICT intervention programmes on body composition and cardiometabolic health in sedentary adolescents. It was hypothesized that HIIT and MICT would significantly improve body composition in terms of body mass index, fat mass percentage, visceral fat area, and waist/hip ratio in sedentary youth compared to a control group. Our secondary hypothesis was that cardiovascular metabolic outcomes benefits in terms of blood pressure, total cholesterol, triglyceride, low-density lipoprotein cholesterol, and HOMA-IR would decrease in HIIT group and MICT group compared to the control group. The present experiment demonstrated the primary hypothesis that HIIT and MICT have similar effects on body composition in sedentary Chinese adolescents. Additionally, the secondary hypothesis was partially confirmed, while both intervention programmes improved cardiometabolic health, the 8-week HIIT was highly effective in increasing cardiometabolic health compared to MICT.

Our findings indicated that the BMI of all groups did not significantly change after the 8-week exercise intervention (*p* > 0.05). Previous studies have presented positive conclusions regarding the effect of HIIT on BMI. 12-week HIIT or 8-week HIIT combined nutrition intervention significantly decreased BMI in obese adolescent boys (age 11.2 ± 0.7 years) (Meng et al., 2022) and overweight adolescent girls (age 15.5 ± 0.7 years) (Bogataj et al., 2021). To our knowledge, however, there was little evidence to confirm the positive findings of BMI after HIIT intervention in normal-weight adolescents. Our data confirm the conclusion of the previous meta-regression analysis, which found that having an entire population classified as overweight or obese significantly moderated the results for BMI (n = 9, β = −1.38, *p* < 0.0001) and waist circumference (n = 7, β = −0.56, *p* = 0.009) (Duncombe et al., 2022). Adolescents who are overweight and obese are associated with significantly increased risk of later cardiometabolic morbidity, such as hypertension, ischaemic heart disease, and stroke in adulthood (Reilly and Kelly, 2011). BMI and WHR are useful measurable indices for assessing obesity and overweight. It should be noted that although the correlation of BMI is associated with increased mortality from cardiovascular disease (CVD), the validity of BMI in predicting CVD is controversial (Westphal, 2008). This disparity of risk may relate to different factors, e.g., body fat and visceral adipose tissue. For example, visceral adipose tissue can predict the development of obesity-related cardiometabolic disease and is an independent predictor of all-cause mortality in men independently of age, race, and sex (Kuk et al., 2006). Body fat mass and visceral adipose will be discussed in a subsequent section of this article. A significant interaction effect between group and time was observed for WHR (*p* < 0.05, effect size 0.35). Follow up for this interaction indicated WHR significantly decreased in HIIT group (*p* < 0.05, effect size 0.43). However, no significant difference was found in MICT group (*p* > 0.05, effect size 0.33) and Control group (*p* > 0.05, effect size 0.37) between baseline and post-intervention. In line with our results, Ahmadi et al. found that 8-week HIIT with nutritional recommendations significantly reduced WHR in obese adolescents (Ahmadi et al., 2020). In contrast, Chuensiri et al. reported that a 12-week HIIT did not affect WHR, whereas novel cardiovascular factors, i.e., carotid intima-media thickness and endothelium-dependent vasodilation improved significantly (*p* < 0.05) in obese preadolescent boys (Chuensiri et al., 2018). Except for the heterogeneity of the samples, we assumed that HIIT combined with the nutritional recommendation was associated with favorable observation in WHR. Future studies are needed to validate these findings, i.e., the effect of HIIT protocol combined with nutritional recommendations on the reduction of lipid profile and other CVD risk factors in children and adolescents.

Body composition, especially fat mass index, is associated with wide range of cardiovascular conditions (Larsson et al., 2020). Our findings demonstrated that HIIT and MICT have small to medium effects on BF (*p* < 0.05, effect size 0.19 and 0.30, respectively), BFP (*p* < 0.05, effect size 0.17 and 0.28, respectively), and VFA (*p* < 0.01, effect size 0.35 and 0.49, respectively) in sedentary Chinese adolescents. In line with our results, previous investigation demonstrated that a 12-week school-based HIIT protocol effectively reduced BF, BFP, and VFA of obese children (age 11.0 ± 0.6 years; BMI 23.6 ± 1.5) (Cao et al., 2022). It is inconsistent with the results of Camacho-Cardenosa et al. (2016) who reported that HIIT group (age 11.0 ± 0.2 years; BMI 18.4 ± 2.8 kg) and MICT group (age 11.2 ± 0.4 years; BMI 20.0 ± 3.3 kg) did not significantly change in total and trunk fat mass after an 8-week high-intensity program developed in adolescents during physical education classes (Camacho-Cardenosa et al., 2016). Furthermore, Lambrick et al. (2016) found that a 6-week high-intensity games intervention induced positive changes in waist circumference for obese participants (age 11.6 ± 0.8 years; BMI 49.3 ± 8.9 kg), however, with no significant difference for normal-weight participants (age 12.3 ± 0.9 years; BMI 32.5 ± 8.9 kg). According to the above findings, we inferred that at least 8-week intervention duration was essential factor to elicit metabolic outcomes in normal-weight adolescents.

Favorable metabolic outcomes were observed in SBP (*p* = 0.018, effect size 0.84), DBP (*p* < 0.01, effect size 1.76), and TG (*p* < 0.01, effect size 1.33) after the 8-week HIIT sessions. Our findings indicated consistency with previous investigation that demonstrated HIIT attenuated SBP and DBP (Alvarez et al., 2017; Ketelhut et al., 2020), TG (Racil et al., 2013; Khammassi et al., 2018). Interestingly, Khammassi et al. (2018) found that variables of findings depend on the patients’ initial obesity degree. So, further studies are then needed to explore this potential implication of HIIT programme in normal-weight adolescents. In contrast, McDaniel et al. demonstrated that 5-week aquatic HIIT induced a trend (*p* = 0.053) for a reduction in SBP, whereas there was no change in DBP (McDaniel et al., 2020). Popowczak et al. (2022) reported that a 10-week HIIT significantly reduced SBP, whereas no impact on DBP. Furthermore, Dias et al. reported that a 12-week HIIT was highly effective in increasing cardiorespiratory fitness, there were no concomitant reductions in blood biomarkers (Dias et al., 2018). Although the blood pressure success change is partly explained by traditional CVD risk factors. However, the release and bioactivity of endothelium-derived nitric oxide induced by shear stress associated with HIIT regulate vascular tone by stimulating guanylate cyclase in the underlying smooth muscle, which may explain the favorable outcome of the blood pressure parameter (Bond et al., 2015; da Silva et al., 2020; Paniagua et al., 2001). No significant differences were observed in TC, HDL-C, and LDL-C in this trial between baseline and post-intervention (*p* > 0.05). Given that SBP, DBP, and TG decreased in HIIT group, there was no significant change in MICT group and Control group in the present study. Our finding demonstrated that the 8-week HIIT intervention programme was highly effective in increasing cardiometabolic health compared to MICT.

Various methods are used to assess insulin sensitivity. However, validity, reproducibility, cost, and degree of subject burden are important factors for researchers to consider when weighing the merits of a particular method (Patarrão et al., 2014). In this study, simple surrogate indexes for insulin resistance are assessed that are derived from blood insulin and glucose concentrations under fasting conditions. Compared with some previous research, e.g., a single bout of HIIT (Cockcroft et al., 2018), 6 weeks of HIIT (Alvarez et al., 2017), and 12 weeks of HIIT (Ryan et al., 2020) both can induce improvements in insulin sensitivity. In this 8-week HIIT trial, statistical results did not confirm an effective change, although there was a slight decrease in insulin resistance (5.63%) in the HIIT group after 8-week intervention, but an increase in the MICT (21.28%) and Control (55.17%). Insulin resistance is a hallmark of obesity and cardiovascular diseases, especially as insulin resistance precedes and contributes to the development of many metabolic disorders, e.g., stroke and atherosclerotic (Nigro et al., 2006; Ago et al., 2018). Furthermore, the evidence showed that insulin resistance was relevant to an increase in different inflammatory markers (Dandona et al., 2004). The increase of inflammatory factors and the associated alterations of oxidative stress seem to play a crucial role in the early stages of atherogenesis (Giannini et al., 2008). It is believed to be involved throughout the atherogenic process, facilitating everything from the initial recruitment of leukocytes to the arterial wall to the eventual rupture of the plaque (Clearfield, 2005). Thus, hs-CRP is a potential adjunct for global risk assessment in the primary prevention of CVD. Our findings indicated that the hs-CRP decreased in the HIIT group (22.44%) and MICT group (19.32%) after the 8-week HIIT intervention. However, a similar decrease was observed in Control group (24.60%). Our findings are not in accordance with previous observations. Paahoo et al. (2021) reported that a 12-week HIIT had more positive effects than aerobic exercise on hs-CRP in obese adolescent boys. Furthermore, a significant hs-CRP decrease was observed in adolescent girls with obesity after 12-week HIIT combined with diet intervention (Playsic et al., 2020). Overall, our results refute the widely reported increase in insulin sensitivity and decrease in hs-CRP demonstrated with HIIT, which is likely due to day-to-day variability in determinations of plasma biomarkers, as well as the heterogeneity of our sample. However, considering that the HIIT intervention programme only marginally reduced insulin resistance, and insulin resistance of MICT group and control group increased, although the MICT group underwent regular training. These results demonstrated that adolescents who experience chronic sedentary behavior, such as taking China’s college entrance exam, remain at high risk of metabolic syndrome after entering university, and these findings reinforce the need to develop programs for the effective prevention of chronic disease in this cohort.

Effective protocols to improve the metabolic profile in adolescents include 10-s sprints interspersed with 10-s recovery (Baquet et al., 2001), 30-s sprints interspersed with 30-s rest bouts (Buchan et al., 2012), and 60-s sprints with 180-s of active recovery between bouts (Corte de Araujo et al., 2012). These protocols are usually practised at 90% maximum heart rate or at 100% peak velocity. Our research adopts multiple times and low-volume in a ratio of 1:3 for all-out sprints and intervals, mainly to reflect better enjoyment and feasibility, so that it is easier for the participants to persist in HIIT for long-term exercise and ensure a high-speed running performance (Williams et al., 2000; Corte de Araujo et al., 2012; Malone et al., 2019). Furthermore, this experiment was conducted strictly to ensure the accuracy and feasibility of the HIIT and MICT programs, and the participants’ subject feelings were good. The heart rate monitoring in this experiment found that the heart rate during the active intervals in the HIIT group and the heart rate during the stabilization period in the MICT group were similar with previous trial delivered in real-world (Camacho-Cardenosa et al., 2016; Logan et al., 2016). Our study enhanced the understanding for confirming the heart rate range for HIIT or MICT delivered on an athletics field.

Despite evidence indicating that HIIT is a time-efficient strategy to improve cardiometabolic risk factors, HIIT appears to promote superior improvements in some cardiometabolic risk factors when performed by healthy participants for at least 8–12 weeks (Kessler et al., 2012). This is consistent with only parts of the cardiometabolic risk factors being examined in our trial. Previous studies also confirmed the inference of sufficient duration (Hottenrott et al., 2012; Racil et al., 2013; Abassi et al., 2020; Playsic et al., 2020). Therefore, it is possible that our intervention was of insufficient duration to improve some health outcomes. Future studies and practitioners may choose to adapt these effective protocols or create new training programs for youth (Chmura et al., 2023; Liu et al., 2024). However, it should be noted that the majority of studies originate from laboratories rather than using a field-based approach, thus in the context of a school-based HIIT experimental studies are highly recommended and needed (Leahy et al., 2019; Kennedy et al., 2020). Our study does highlight the possibility and feasibility of sprint interval training to induce favorable outcomes in a university setting.

The study has several limitations. The study does not provide enough evidence to interpret the mechanism of sedentary causes and potential increased risk of insulin resistance, including whether it is related to gut health caused by food intake. Additionally, despite the favorable metabolic outcomes observed, a small sample limits this study’s generalization ability to a broader population. Follow up research should refer to sex, nutritional control, and gut bacterial monitoring when designing the study. Therefore, the potential impact of multiple factors able to influence blood parameters was minimized.

## Conclusion

This study examined the physiological efficacy of HIIT compared with MICT in sedentary adolescents, and our findings demonstrated that 8-week HIIT and MICT have similarly favorable outcomes in body fat mass, body fat percentage, and visceral fat area. HIIT can decrease waist/hip ratio, systolic blood pressure, diastolic blood pressure, and triglyceride, but not MICT. We provide an update on body composition and blood biochemical parameters responses after HIIT and MICT in sedentary Chinese adolescents; we propose that HIIT is an efficacious part of this cohort management. Furthermore, an 8-week HIIT and MICT may have limited scope to decrease insulin resistance in sedentary normal-weight adolescents. The precise determination of the dose-response between different protocols and health-related outcomes is worth further exploring so as to optimize cardiometabolic benefits.

## Data Availability

The raw data supporting the conclusions of this article will be made available by the authors, without undue reservation.
